# The prevention of musculoskeletal injuries in volleyball: the systematic development of an intervention and its feasibility

**DOI:** 10.1186/s40621-017-0122-y

**Published:** 2017-09-01

**Authors:** Vincent Gouttebarge, Marije van Sluis, Evert Verhagen, Johannes Zwerver

**Affiliations:** 1 Dutch Consumer Safety Institute, Overschiestraat 65, 1062 XD Amsterdam, The Netherlands; 2 Academic Center for Evidence based Sports medicine (ACES), Academic Medical Center, Amsterdam, The Netherlands; 3 Amsterdam Collaboration for Health & Safety in Sports (ACHSS), IOC Research Center, Academic Medical Center / VU University Medical Center , Amsterdam, The Netherlands; 4 Division of Exercise Science and Sports Medicine, University of Cape Town, Cape Town, South Africa; 5 Center for Sport and Exercise Medicine, University of Groningen, University Medical Center Groningen, Groningen, The Netherlands; 6 Department of Public and Occupational Health, VU University Medical Center, Amsterdam, The Netherlands; 7 Australian Centre for Research into Injury in Sport and its Prevention (ACRISP), Federation University Australia, Ballarat, Australia

**Keywords:** Volleyball, Musculoskeletal injuries, Prevention, Intervention mapping

## Abstract

**Background:**

A scientific research project has started in the Netherlands with the aim of developing and implementing an evidence-based intervention to prevent the occurrence of musculoskeletal injuries among young and adult recreational volleyball players. This article describes (i) the systematic development of the intervention; and (ii) the assessment of its feasibility in terms of relevancy, suitability and usability.

**Systematic development of the intervention:**

The development of the intervention was based on the Intervention Mapping structured and systematic process. First, the needs assessment conducted among the main actors within recreational volleyball revealed that an intervention was needed for injury prevention, ideally embedded prior to a volleyball activity (training or match) within the warm-up, delivered by trainers/coaches, and available in an application for smartphone/tablet or website. Second, the objective and target groups of the intervention were defined, namely to prevent or reduce the occurrence of finger/wrist, shoulder, knee and ankle injuries among both young and adult recreational volleyball players. Third, preventive measures and strategies (e.g. core stability, strength and balance) were selected in order to accomplish a decrease in injury incidence. Last, the intervention ‘VolleyVeilig’ was finally developed, a warm-up programme including more than 50 distinct exercises and lasting 15 min.

**Feasibility of the intervention:**

A quasi-experimental research based on a one-group post-test design was conducted over a period of 3 weeks among 41 volleyball players and five coaches from five adult recreational teams, who were asked to use the intervention. Degree of relevancy, suitability and usability of the warm-up programme ‘VolleyVeilig’ were measured among players and coaches on an 11-point scale (varying from ‘completely disagree’ to ‘completely agree’).

All groups of exercises within the warm-up programme were positively assessed with regard to their relevancy, suitability and usability, mean scores ranging from 7.7 to 8.3. Group interviews revealed especially that the warm-up programme in its current form was not suitable as a pre-match warm-up.

**Conclusion:**

The warm-up programme ‘VolleyVeilig’ developed in order to prevent or reduce the occurrence of musculoskeletal injuries in recreational volleyball was positively assessed by volleyball players and coaches with regard to its relevancy, suitability and usability. Before its nationwide implementation, the effectiveness of the intervention on injury reduction among volleyball players should be conducted.

## Background

Volleyball is one of the big five international sports being played today, relying on 220 affiliated national federations with an estimate of 800 million players who play volleyball at least once a week (Fédération Internationale de Volleyball 2016). While being undoubtedly beneficial to physical, mental and social well-being, volleyball, like other sports, is also associated with a risk for musculoskeletal injuries. The incidence of musculoskeletal injuries among volleyball players ranges from 1.7 to 10.7 injuries per 1000 playing hours, occurring more often during matches and among male players (Bahr & Bahr 1997; Kilic et al. 2017). In the Netherlands, similar incidence rates are reported annually, reaching up to 5.7 injuries per 1000 playing hours in 2014 (Dutch Consumer Safety Institute 2015). Musculoskeletal injuries among volleyball players can be either acute or overuse injuries, and occur principally in the fingers/wrists, ankles, shoulders and knees (Dutch Consumer Safety Institute 2015; Kilic et al. 2017). These injuries lead to substantial direct and indirect healthcare costs and are likely to induce impairments in daily life, sport and/or work (Verhagen et al. 2005). With regard to the high incidence of musculoskeletal injuries among volleyball players and its negative consequences and societal impact, one might expect effective preventive measures to be available, especially for the common finger/wrist, shoulder, knee and ankle injuries.

However, volleyball-specific preventive measures remain scarce in the Netherlands. This was demonstrated in a recent study of the Dutch Volleyball Federation (Nevobo) in which volleyball players emphasised their need for more attention and specific measures regarding injury prevention (Elling et al. 2014). Consequently, a scientific research project was started in the Netherlands with the aim of developing and implementing an evidence-based volleyball-specific intervention to prevent the occurrence of musculoskeletal injuries among young and adult recreational volleyball players. This article describes subsequently: (i) the systematic development of the intervention; and (ii) the assessment of its feasibility (in terms of relevancy, suitability and usability) in recreational adult volleyball teams.

### The systematic development of the intervention

In health promotion research, interventions have been developed according to Intervention Mapping (IM), a structured and systematic process often applied in this field of health research that can also be used within sport injury prevention research (Bartholomew et al. 1998; Kok et al. 2016; Verhagen et al. 2014). The IM process relies on six consecutive steps, namely: 1) needs assessment; 2) formulating the objectives and target group(s) of the intervention; 3) selection of theories and/or practical strategies for the intervention; 4) development of the intervention; 5) development of an adoption and implementation plan; 6) development of an evaluation plan (Bartholomew et al. 1998; Kok et al. 2016). Steps 5 and 6 of the IM process are typically assessed through a thorough evaluation (effectiveness and implementation) of the intervention. This evaluation (effectiveness and - in case the intervention appears to be effective- implementation) has already been planned for the upcoming years. To strictly develop an intervention aiming at the prevention of musculoskeletal injuries among recreational young and adult volleyball players, steps 1 to 4 of the IM structured and systematic process were applied.

#### Needs assessment (step 1 of the IM process)

The key purposes of the needs assessment were: (i) to assess the needs (content, form, behaviour) and support for the intervention; (ii) to identify the objectives and target groups of the intervention; and (iii) to discuss strategies for the implementation of the intervention. Therefore, two semi-structured focus-group interviews were held with the 12 to 16 main actors within recreational volleyball, namely players, trainers/coaches, (para-)medics, policy makers and representatives of clubs, and policy makers and representatives of the Nevobo. Each focus-group interview took 1 hours (with a 10-min break) and was held in April 2016. Subsequently, an anonymous electronic survey (in Dutch) was set up and distributed by Nevobo (June 2016) to volleyball players and trainers/coaches in order to explore whether the qualitative information gathered through the focus-group interviews was largely supported. Fifteen questions were formulated in relation, among others, to: (i) current and future injury prevention behaviour; (ii) needs and support for the intervention; (iii) characteristics (content, form) of the intervention; and (iv) strategies to deliver the intervention.

Information collected through the focus-group interviews indicated that: (i) injuries among young and adult recreational volleyball players occur principally in the fingers/wrists, shoulders, knees and ankles; (ii) support measures or interventions concerning injury prevention were lacking in clubs; (iii) there was a need for an injury preventive intervention for both young and adult recreational volleyball players; (iv) an injury preventive intervention could be embedded prior to a volleyball activity (training or match) within the warm-up; (v) the injury preventive intervention could be delivered by trainers/coaches; (vi) information and instructions about the injury preventive intervention could be presented in an application for smartphone/tablet or website. The aforementioned qualitative information was confirmed by the outcomes of the electronic survey completed by nearly 2000 volleyball players (95%) and trainers/coaches (5%). The main outcomes of the survey are presented in Table 1.

**Table 1:** Needs assessment towards an injury preventive intervention in volleyball

*Current and future injury prevention behaviour*
▪ 30% of the respondents mentioned that there was no attention given to injury prevention in their clubs.
▪ 55% of the respondents indicated that their knowledge and capacities towards injury prevention was insufficient.
▪ 95% of the respondents stated that any attention for injury prevention should be given within the warm-up prior a volleyball activity.
*Needs and support for an injury preventive intervention*
▪ 95% of the respondents indicated that the intervention was needed for both young and adult recreational volleyball players.
*Characteristics of an injury preventive intervention*
▪ 92% of the respondents mentioned that the intervention for young and adult recreational volleyball players should be embedded within the warm-up prior a volleyball activity (training or match).
▪ 25%, 44% and 22% indicated that they were likely to use the intervention once, twice and three times a week, respectively.
▪ 43% and 36% of the respondents stated that they were likely to spend each time 10 and 15 min, respectively, to the intervention.
*Strategies to deliver an injury preventive intervention*
▪ Two-third of the respondents indicated that the intervention needed to be delivered through an application for smartphone/tablet and website.
▪ The majority of the respondents stated that the content of the intervention should be principally with videos by a trainer/coach (62%), a physical therapist (55%) or an elite volleyball player (52%).

#### Objective and target group(s) of the intervention (step 2 of the IM process)

Experts in volleyball (players, trainers/coaches, club representatives) and/or injury prevention (strength and conditioning trainers, physical therapists, sports physicians) were approached in order to formulate clearly the objective of the intervention and specify its target groups. For this purpose, a meeting was held during which information gathered through the needs assessment (focus-group interviews and survey) was used, as well as the findings of a recent systematic literature review on the incidence, prevalence, etiology and preventive measures of volleyball injuries (Kilic et al. 2017). The following intervention objective and target groups were formulated, namely to prevent or reduce the occurrence of finger/wrist, shoulder, knee and ankle injuries among both young and adult recreational volleyball players.

#### Selection of preventive measures and strategies for the intervention (step 3 of the IM process)

Two multidisciplinary expert meetings were held in order to select preventive measures (content) and strategies (form) for the intervention. A total of 15 participants were selected with regard to their expertise and role in volleyball (players, trainers/coaches, club representatives) and/or injury prevention (strength and conditioning trainers, physical therapists, sports physicians). Relevant preventive measures (content) for the intervention were selected accordingly to the Van Mechelen’s ‘sequence of prevention’ model (four steps related to incidence, etiology and effective prevention of sports injuries (Van Mechelen et al. 1992)). For this purpose, information gathered through the needs assessment (focus-group interviews and survey) was used, as well as the findings of a recent systematic literature review on the incidence, prevalence, etiology and preventive measures of volleyball injuries (Kilic et al. 2017). Led by an experienced researcher, the experts’ meetings were audiotaped and notes were taken. Each expert meeting took 3 hours (with a 10-min break). Both multidisciplinary expert meetings were held in June and July 2016.

Experts identified preventive measures (content) directed towards the modifiable risk factors of shoulder, knee and ankle injuries. For instance, experts looked closely at a proprioceptive balance board training programme that was found to be effective in preventing ankle sprains among volleyball players as well as a preventive programme that was found to be effective in reducing shoulder injuries among handball players (Andersson et al. 2016; Verhagen et al. 2004). Experts discussed the identified preventive measures and consensus between experts was reached for the selection of preventive measures. The key preventive measures and strategies selected by the experts for the prevention of shoulder, knee and ankle injuries were related to core stability, strength, coordination and balance, and mobility, i.e. flexibility. Due to the lack of scientific evidence for the primary prevention of finger/wrist injuries, experts chose to include advice about taping (secondary/tertiary prevention). In accordance to the needs of volleyball players and trainers/coaches, experts agreed that all selected preventive measures (content) should be embedded within the warm-up prior to a volleyball activity (training or match) and made available through a website and an application for smartphone/tablet.

#### Development of the intervention (step 4 of the IM process)

All information gathered in the previous steps were synthetized and translated for the development of the intervention. For this purpose, three work sessions with four experts in the field of volleyball and injury prevention (player, physical therapist, strength and conditioning trainer) were held. Led by an experienced researcher, each work session took 4 hours (with three breaks of 10 min). The three work sessions were held in July and September 2016.

The intervention (in Dutch) consists of a warm-up programme including more than 50 distinct exercises (with different variations and levels for young and adult players) that has to be used at least twice a week prior to any volleyball activity (training or match). The intervention is divided into six phases of 5 to 6 weeks (in line with a typical volleyball season), providing volleyball trainers/coaches every week with a new warm-up programme that shows progressive increments in terms of intensity, frequency, duration and/or complexity. Each warm-up programme lasts 15 min and is divided into a preparatory cardiovascular warm-up (2 to 3 minutes), core stability exercises (2 to 3 minutes), exercises principally directed at preventing knee injuries (4 to 5 minutes), and exercises principally directed towards preventing shoulder injuries (4 to 5 minutes). The exercises directed towards preventing ankle injuries are integrated within each stage of the warm-up programme. All warm-up programmes and related exercises of the intervention are embedded into a website and an application for smartphone/tablet (automatic synchronisation) that are accessible with a user name and password by trainers/coaches. Information and instructions about the exercises are available as texts and videos (including voice-over). Advice about taping fingers/wrists is available within the application for smartphone/tablet. Trainers/coaches are informed and instructed about the warm-up programme and related exercises during a one-hour training session (delivered by an injury prevention consultant). Illustrations of the website and application for smartphone/tablet, as well as examples of exercises within the intervention are presented in Appendix.

#### The feasibility of the intervention

The objective was to explore the feasibility (in terms of relevancy, suitability and usability) of the developed intervention among recreational adult volleyball players.

## Methods

A quasi-experimental research based on a one-group post-test design was conducted over a period of 3 weeks (Bowen et al. 2009). This study did not meet the criteria for the Medical Research Involving Human Subjects Act and therefore did not require approval from a Dutch human ethics research committee. This study was conducted in accordance with the Declaration of Helsinki and the Personal Data Protection Act.

The participants were healthy recreational adult volleyball players and their coaches. Inclusion criteria for players were: (i) 18 years of age or older; (ii) playing in a volleyball team competing recreationally; (iii) training and/or competing at least three times a week; (iv) speaking and reading Dutch fluently. Inclusion criteria for coaches were: (i) 18 years of age or older; (ii) speaking and reading Dutch fluently. As it concerns a ‘feasibility study’, a convenient sample of at least 30 volleyball players and three coaches from three teams (8–10 players and one coach per team on average) of three different clubs was to be recruited (Bowen et al. 2009).

For each exercise within the intervention, the following outcomes (assessed among players and coaches) were measured: (i) degree of relevancy, i.e. added value of the exercises; (ii) degree of suitability of the exercises within training sessions; (iii) degree of usability of the exercises. For this purpose, questions were formulated, for instance: “Is this exercise relevant and of added value within the warm-up of volleyball players?”; “Are the instructions for this exercise clear and complete?”; “Can this exercise be performed by volleyball players?”. All outcomes were measured on an 11-point scale (varying from ‘completely disagree’ to ‘completely agree’). Players and coaches were informed by the Nevobo by email about the intervention and related feasibility study (aim and procedures). If willing to participate voluntarily in the study, players and coaches signed an informed consent. The different weekly warm-up programmes (and related core elements, i.e. exercises) were divided over the different teams, and players and their coaches were asked to use a new weekly warm-up programme at every session (training or competition) during a period of 3 weeks. After using a new warm-up programme, players and coaches were asked to complete a paper questionnaire including all outcomes under study. After the whole period of 3 weeks, a semi-structured face-to-face group interview (15 min) was held in each club with players and coaches about the feasibility of the intervention.

All data were entered within the statistical software IBM SPSS Statistics 23.0 for Windows. For each outcome and group of exercises, descriptive data analyses were conducted. In addition, outcomes on an 11-point scale were transformed as a dichotomous variable, with a score of six or more being interpreted as being positive in favour of a group of exercises.

## Results

In total, 41 volleyball players and five coaches from five teams (three different clubs from the regional levels) were enrolled in the study. All groups of exercises within the warm-up programme ‘VolleyVeilig’ were positively assessed by the volleyball players and coaches. For all exercises, the mean degree of relevancy was 7.7 (SD = 0.9; min-max = 6.3–9.0), the mean degree of suitability was 8.3 (SD = 0.85; min-max = 7.0–9.5), and the mean degree of usability was 8.1 (SD = 0.7; min-max = 7.0–9.5). The group interviews confirmed that: (i) players and coaches were very pleased with the six phases of 5 to 6 weeks as this coincides with the regular periodization of a volleyball season; (ii) the videos and texts describing the exercises of the warm-up programme were necessary for their optimal execution; and (iii) the warm-up programme could be executed within the intended time of 15 min. The group interviews also revealed that: (i) an explanation of the purpose of the exercises was necessary; and (ii) the warm-up programme in its current form was not suitable as pre-match warm-up (too demanding).

## Discussion

This article aimed to describe: (i) the systematic development; and (ii) the assessment of the feasibility of an intervention aiming to prevent or reduce the occurrence of fingers/wrists, ankle, shoulder and knee injuries among both young and adult recreational volleyball players. The systematic development of the intervention was based on parts of the IM process, involving all main actors within recreational volleyball. As a result, the warm-up programme ‘VolleyVeilig’ was developed, including more than 50 distinct exercises and lasting 15 min. All groups of exercises within ‘VolleyVeilig’ were positively assessed with regard to their relevancy, suitability and usability by volleyball players and coaches.

### Injury prevention programmes across sports

As sports injuries can have negative influences on sport participation and might result in high costs for society, exercise-based interventions to prevent or reduce multiple (location and type) injuries have been developed across several sports. In South Africa, the BokSmart Safe Six intervention for rugby was developed in order to prevent injuries in the most commonly injured areas (knee, hamstring, lower limb, ankle and shoulder) (Sewry et al. 2016). Based on easy-to-perform exercises to increase strength, joint stability, balance and control, the BokSmart Safe Six intervention can be performed any place, any time, without requiring any equipment or facility, and is of a short enough duration that it does not interfere with regular training (Sewry et al. 2016). Also in rugby, a recent study showed that a preventive movement control exercise programme reduced match injury outcomes among young rugby players (Hislop et al. 2017). In the Netherlands, an integral exercise-based intervention (more than 50 exercises; age- and gender-specific) was developed to prevent musculoskeletal injuries in the lower limbs among youth/adult hockey players (Gouttebarge & Zuidema 2017). An effectiveness study is still ongoing, but this specific hockey warm-up programme showed to have a moderate effect on the level of knowledge and skills of hockey coaches/trainers about injury prevention (Gouttebarge & Zuidema 2017). The Fédération Internationale de Football Association (FIFA) has developed and promoted the FIFA 11+ injury prevention programme (Bizzini & Dvorak 2015). Divided into three parts and based on 15 exercises, the FIFA11+ is a warm-up programme focussing on injury prevention among football players aged 14 and older (Bizzini & Dvorak 2015). Several randomized controlled trials have evaluated the programme, showing that the FIFA11+ reduces injuries by 35% among female players and by 50% among male and youth players (Bizzini & Dvorak 2015). Considering the conceptual similarities between the aforementioned programmes and the warm-up programme ‘VolleyVeilig’, and with regard to the 35–50% reduction achieved with the FIFA11+, one might be confident that the use of the ‘VolleyVeilig’ is likely to lead to the prevention or reduction of finger/wrist, shoulder, knee and ankle injuries in recreational volleyball.

### Future directions

As indicated through our feasibility study, a warm-up programme suitable as pre-match warm-up should be designed (being shorter and lighter), while an explanation of the purpose of the exercises should be added. Then, in accordance with the fourth step within the Van Mechelen’s ‘sequence of prevention’ model, the systematic development of ‘VolleyVeilig’ as an intervention to prevent the occurrence of musculoskeletal injuries among recreational volleyball players should be followed by an evaluation of its effectiveness (Van Mechelen et al. 1992). To this end, a randomized prospective controlled trial will be conducted in the Netherlands during the 2017–2018 volleyball season among 640 recreational adult volleyball players (Gouttebarge et al. 2017). If the warm-up programme ‘VolleyVeilig’ is found to prevent or reduce injuries in a controlled setting, its impact in a real-world sport setting will be evaluated according to the Reach Efficacy Adoption Implementation Maintenance (RE-AIM) framework. Originally designed by Glasgow et al. (1999) to evaluate the public health impact of health promotion interventions, the RE-AIM framework has been used to optimally translate research into practice (Glasgow et al. 1999). A clear understanding in the barriers and facilitators for all five RE-AIM dimensions remains a prerequisite for a successful implementation and for a significant impact among volleyball players.

## Conclusions

The warm-up programme ‘VolleyVeilig’ which has been developed in order to prevent or reduce the occurrence of injuries in fingers/wrists, ankles shoulders and knees in recreational volleyball, was positively assessed by volleyball players and coaches with regard to its relevancy, suitability and usability. Before its nationwide implementation, the effectiveness of the intervention on injury reduction among volleyball players should be conducted.

## Figures and Tables

**Fig. 1:**
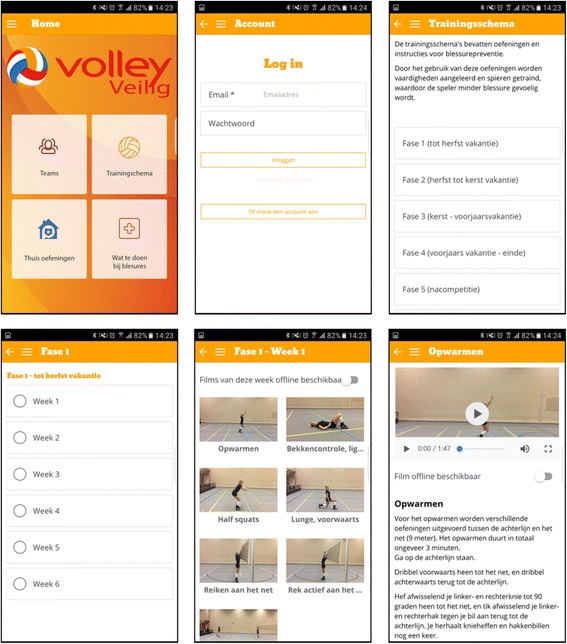
The intervention for volleyball players

## Abbreviations

FIFA: Fédération Internationale de Football Association
IM: Intervention Mapping
Nevobo: Dutch Volleyball Federation
RE-AIM: Reach Efficacy Adoption Implementation Maintenance
